# Detection of the blaNDM-1 Gene in Carbapenem-Resistant Enterobacterales Causing Urinary Tract Infections in Patients at a Rural Teaching Hospital

**DOI:** 10.7759/cureus.81811

**Published:** 2025-04-06

**Authors:** Ajay Kumar, Amisha Sharma, Priya Mehrishi, Seema Solanki, Sameer Singh Faujdar, Ashma Khatun

**Affiliations:** 1 Medical Microbiology, Maharishi Markandeshwar Medical College and Hospital, Solan, IND; 2 Microbiology, Maharishi Markandeshwar Medical College and Hospital, Solan, IND

**Keywords:** carbapenemase, enterobacterales, mcim, mcnp, ndm-1, uropathogens

## Abstract

Background: Carbapenem-resistant *Enterobacterales *(CRE) pose a significant public health threat due to their resistance to last-line antibiotics. Urinary tract infections (UTIs) caused by multidrug-resistant organisms have become a major challenge in clinical settings. The spread of CRE is largely attributed to the acquisition of carbapenemase-encoding genes, horizontal gene transfer, and overuse of broad-spectrum antibiotics.

Methodology: A total of 9235 urine samples were analyzed, and more than 10^5^ CFU/mL bacterial count was considered positive for UTI. These bacteria were identified and further screened for CRE and *bla*NDM-1 genes.

Results: A total of 9235 urine samples were analyzed, out of which 555 were identified as *Enterobacterales*. Among these, 47 were confirmed as CRE, accounting for 8.5% of the *Enterobacterales *isolates. Out of 47 CRE, 28 were positive for the *bla*NDM-1 gene.

Conclusions: The study highlights the increasing burden of CRE and the urgent need for stringent antimicrobial stewardship, effective infection control measures, and the development of new therapeutic strategies to combat MDR infections. Additionally, risk factors associated with CRE infections, their implications on public health, and potential future therapeutic approaches are discussed.

## Introduction

Urinary tract infections (UTIs) are prevalent bacterial infections affecting individuals across all ages, accounting for a significant proportion of hospital and community-acquired infections worldwide [1]. UTIs are mainly caused by bacteria, among them *Enterobacterales*, including *Escherichia coli *and *Klebsiella pneumoniae*, being the predominant causative agents. These infections range from uncomplicated cystitis to severe and life-threatening pyelonephritis and urosepsis, particularly in immunocompromised individuals, elderly patients, and those with indwelling catheters [2]. The emergence and rapid dissemination of antimicrobial-resistant bacteria, particularly Carbapenem-resistant *Enterobacterales *(CRE), pose a severe global health challenge. Carbapenems are considered the last line of defense against multidrug-resistant (MDR) Gram-negative bacteria. However, the increasing frequency of carbapenem resistance among uropathogens is driven by multiple factors, including genetic resistance mechanisms, antibiotic misuse, healthcare-associated infections (HAIs), and environmental reservoirs. The primary cause is the acquisition of carbapenemase-producing genes such as KPC, NDM, VIM, and OXA-48, which are often carried by plasmids, enabling rapid spread among bacterial populations. The overuse and misuse of carbapenems and other broad-spectrum antibiotics in both hospital and outpatient settings further accelerate drug resistance. HAIs play a major role, as CRE uropathogens are commonly transmitted in hospitals, nursing homes, and other medical facilities, particularly among patients with prolonged urinary catheters or those undergoing invasive procedures. Poor infection control practices, including inadequate hand hygiene and contamination of medical instruments, increase the risk of infection. Additionally, CRE strains have also been detected in environmental reservoirs such as wastewater, food sources, and animals, which raises concerns about their spread beyond healthcare settings. The combination of these factors underscores the urgent need for robust antimicrobial stewardship, strict infection prevention measures, and ongoing surveillance to curb the rising threat of CRE in UTIs [3,4]. Early detection of CRE and the *bla*NDM-1 gene enables the implementation of strict infection prevention measures, such as patient isolation, contact precautions, enhanced surveillance, and antimicrobial stewardship programs, to limit hospital outbreaks and community spread. Combining phenotypic and genotypic approaches ensures a comprehensive diagnosis, allowing healthcare facilities to effectively manage CRE infections and prevent their transmission [5,6]. This study aims to assess the prevalence of CRE and the presence of *bla*NDM-1 among uropathogens isolated from urine samples and provide insights into the implications of CRE infections in healthcare settings. The findings of this study will help guide infection control policies, antimicrobial stewardship initiatives, and the development of new therapeutic strategies to curb the spread of CRE.

## Materials and methods

Study setting and data collection

This was a prospective and cross-sectional study. It was carried out from June 2022 to May 2024 in the department of Microbiology, Maharishi Markandeshwar Medical College and Hospital, Solan, India, after obtaining ethical clearance from the Institutional Ethics Committee, Maharishi Markandeshwar Medical College and Hospital (MMMCH/IEC/22/589).

Isolation and identification of uropathogens

All urine specimens received in the microbiology laboratory were inoculated on cysteine-lactose-electrolyte-deficient (CLED) agar as per standard protocol, followed by incubation at 37°C for 18-24 hours under aerobic conditions. Bacterial identification was performed by observing colony morphology, followed by Gram staining and the Vitek 2 system. Antimicrobial susceptibility testing was conducted using the Kirby-Bauer disk diffusion method, and results were interpreted according to Clinical and Laboratory Standards Institute (CLSI) guidelines [7].

Detection of CRE

Screening Test

All *Enterobacterales *were further subjected to carbapenem-resistance testing (10 μg imipenem) by Kirby-Bauer disc-diffusion methods. Isolates with the inhibitory zone of ≤19 mm were considered positive for CRE as per CLSI guidelines. These isolates were further subjected to identify the carbapenemase-producing CRE (CP-CRE) using phenotypic methods [7,8].

Phenotypic Methods for the Detection of CP-CRE

Rapidec Carba Nordmann‐Poirel (NP): Rapidec Carba NP is a rapid colorimetric test used for detecting carbapenemase production in *Enterobacterales*, providing results within two hours. It functions by identifying the hydrolysis of imipenem, which leads to a pH change, causing a color shift from red to yellow in positive cases. This test is highly valuable for infection control and antimicrobial resistance surveillance, offering a simple, cost-effective alternative to molecular assays. Rapidec Carba NP effectively detects major carbapenemases (KPC, NDM, VIM, IMP, OXA-48), enabling early identification of CRE and facilitating appropriate antibiotic stewardship [8].

Ethylenediaminetetraacetic acid (EDTA)-imipenem combined disk test: The EDTA combined disc diffusion method was used to detect MBL production. EDTA, a chelating agent, was added to the carbapenem disc to inhibit MBL activity. In this method, Mueller Hinton agar (MHA) plates were lawn cultured with the tested bacterial suspension adjusted to McFarland (MF) 0.5 unit. A carbapenem disc was placed on the agar surface, and an imipenem+EDTA disc was positioned 20-25 mm away from the carbapenem disc for 18-24 hours (37°C). The clearance zone was measured around the carbapenem disc. An upsurge in zone size of the carbapenem disc with EDTA compared to a single carbapenem disc indicated MBL production [9].

Modified carbapenem-inactivation method (MCIM): MCIM was used for the detection of carbapenemase enzymes among *Enterobacterales*. A loopful of the test organism was mixed in TSB to which a meropenem/imipenem disc was added and further incubated for four hours (35-37°C). Following incubation, the disc was taken out from Tryptic Soy Broth and placed on a pre-inoculated MHA plate containing a suspension (0.5 MF) of* E. coli *strain that was sensitive to carbapenem and was incubated for 18-24 hours (37°C), then the zone of inhibition was measured. A significant reduction in the zone of inhibition (≤19 mm) or no zone indicates carbapenemase production by the test organism, while a clear inhibition zone (≥20 mm) indicates the absence of the enzyme [10].

The modified Hodge test: This phenotypic test detects carbapenemase production in *Enterobacterales*. The carbapenem-sensitive *E. coli* strain was set to 0.5 MF and was further diluted to a 1:10 ratio, which was then cultured on MH agar. Imipenem disc (10 µg) was positioned at the middle of the MH agar. Streaking of the test isolate was done from the edge of the disc to the edge of the plate, which was then kept for 16-24 hours (35-37°C). After incubation, a cloverleaf-like indentation at the intersection of the streak line and clearance zone around the carbapenem disc indicated a positive result. A clear, uninterrupted zone of inhibition indicated a negative result [11].

Genotypic Detection

Polymerase chain reaction (PCR) is a highly sensitive and specific molecular technique used to detect the *bla*NDM-1 gene, which encodes “New Delhi Metallo-β-lactamase-1,” a key enzyme responsible for carbapenem resistance in *Enterobacterales *and other Gram-negative bacteria. The detection process involves DNA extraction from the bacterial isolate, amplification of the *bla*NDM-1 gene using specific primers 5′-GGTTTGGCGATCTGGTTTTC-3 (forward), 5′-CGGAATGGCTCATCACGATC-3 (reverse), and visualization of the PCR products via gel electrophoresis or real-time PCR (qPCR) for quantitative analysis. The presence of a target-sized amplicon (e.g., 621 bp for conventional PCR) confirms the presence of *bla*NDM-1.The controls used were *K. pneumoniae* ATCC BAA-2146. The PCR cycling conditions for the detection of the *bla*NDM-1 gene with a 621 bp amplicon typically involve an initial denaturation at 95°C, followed by 30 cycles of denaturation at 95°C for 30 seconds, annealing at 55°C for 30 seconds, and extension at 72°C for 30 seconds. The process concludes with a final extension at 72°C for 10 minutes to ensure complete amplification. Real-time PCR offers additional advantages, such as rapid detection, lower contamination risk, and quantification of gene expression. This method is crucial for epidemiological surveillance, infection control, and guiding antimicrobial therapy in cases of CRE infections [12].

Statistical analysis

Data obtained from phenotypic and genotypic methods were meticulously entered in Excel (Microsoft® Corp., Redmond, WA, USA) and analyzed with IBM SPSS Statistics for Windows, Version 28 (Released 2021; IBM Corp., Armonk, New York, United States). A chi-square test was chosen to obtain frequencies, and percentages were calculated for categorical variables (e.g., prevalence of carbapenem resistance, carbapenemase production by various phenotypic methods, organism distribution, and gene detection). The analyzed data were then presented as percentages and p-values. A result was deemed statistically significant if the probability was less than 0.05.

## Results

A total of 9,235 urine specimens were received, among which 653 (7%) were identified as uropathogens. Of these, 555 (85%) belonged to the *Enterobacterales *group, while 98 (15%) were non-*Enterobacterales*. Among the 555 *Enterobacterales *isolates, *E. coli* was the most prevalent (456 isolates), followed by *Klebsiella* spp. (56), *Citrobacter* spp. (30), *Proteus *spp. (5), *Enterobacter* spp. (5), and a single isolate of each *Salmonella, Morganella*, and *Serratia* species.

A total of 47 isolates (8.46%) were found to be CRE using the Kirby-Bauer disc diffusion method. Among these, *E. coli *(36) was the most common, followed by *Klebsiella* species (5), *Citrobacter* species (4), *Serratia* species (1), and *Morganella* species (1). The highest number of CRE cases was observed in the 41-60 years age group (17 cases), followed by 61-80 years (12 cases) and 21-40 years (11 cases), with the lowest numbers in the extreme age groups of 1-20 years (4 cases) and >81 years (3 cases).

**Figure 1:**
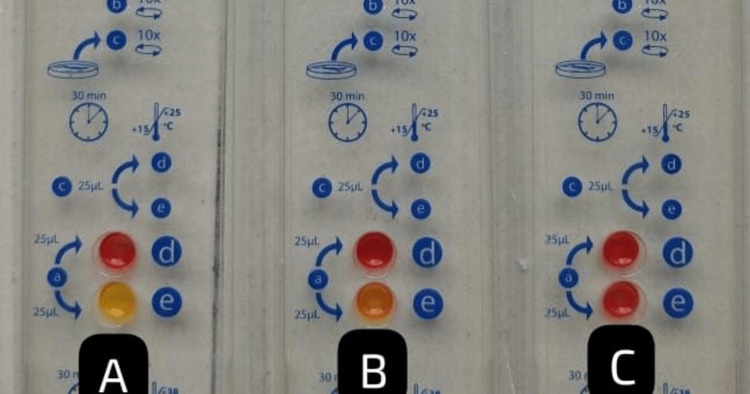
Rapidec Carba NP test (A) Positive control: Well E turns into a yellow color, indicating positive for carbapenemase. (B) Test strain: Well E turns into a yellow color, indicating positive for carbapenemase. (C) Negative control: Well E remains red color, indicating negative for carbapenemase.

**Figure 2:**
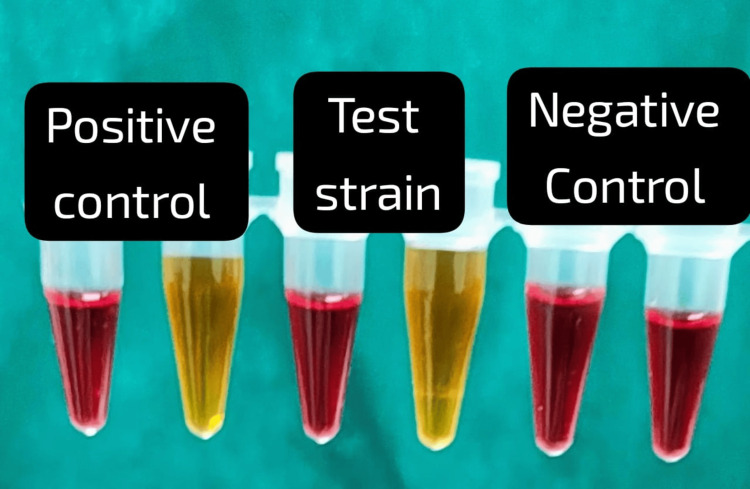
Modified Carba NP test Positive control: Reagent color turns yellow, indicating the presence of carbapenemase. Test strain: Reagent color turns yellow, indicating the presence of carbapenemase. Negative control: Reagent color remains red, indicating the absence of carbapenemase.

**Figure 3:**
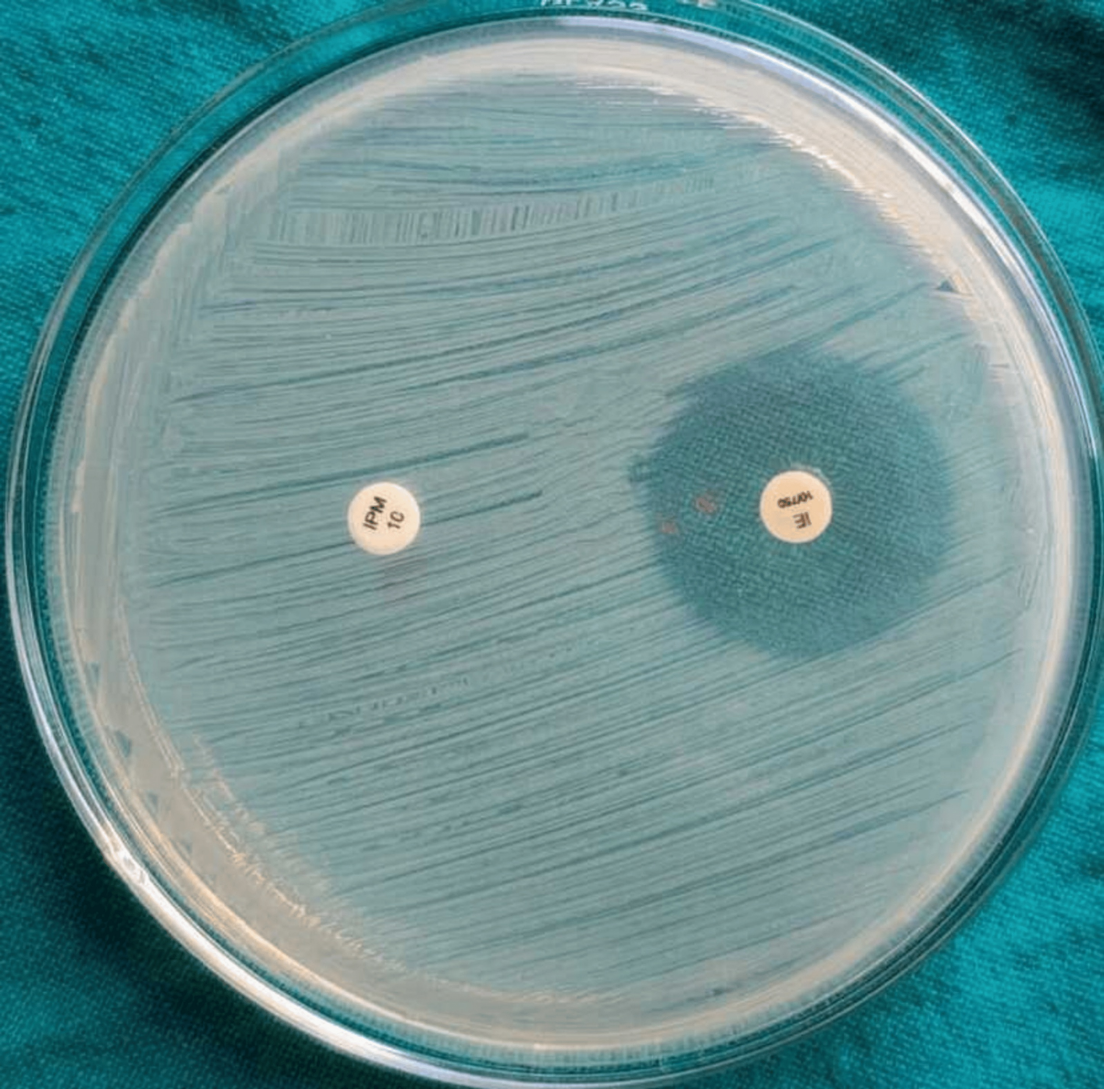
EDTA disc diffusion test An enhanced zone of inhibition around the imipenem-EDTA disc that is ≥ 7 mm larger than that around the imipenem disc alone indicates the presence of MBL-producing isolates. EDTA: ethylenediaminetetraacetic acid; MBL: Metallo-β-lactamase

**Figure 4:**
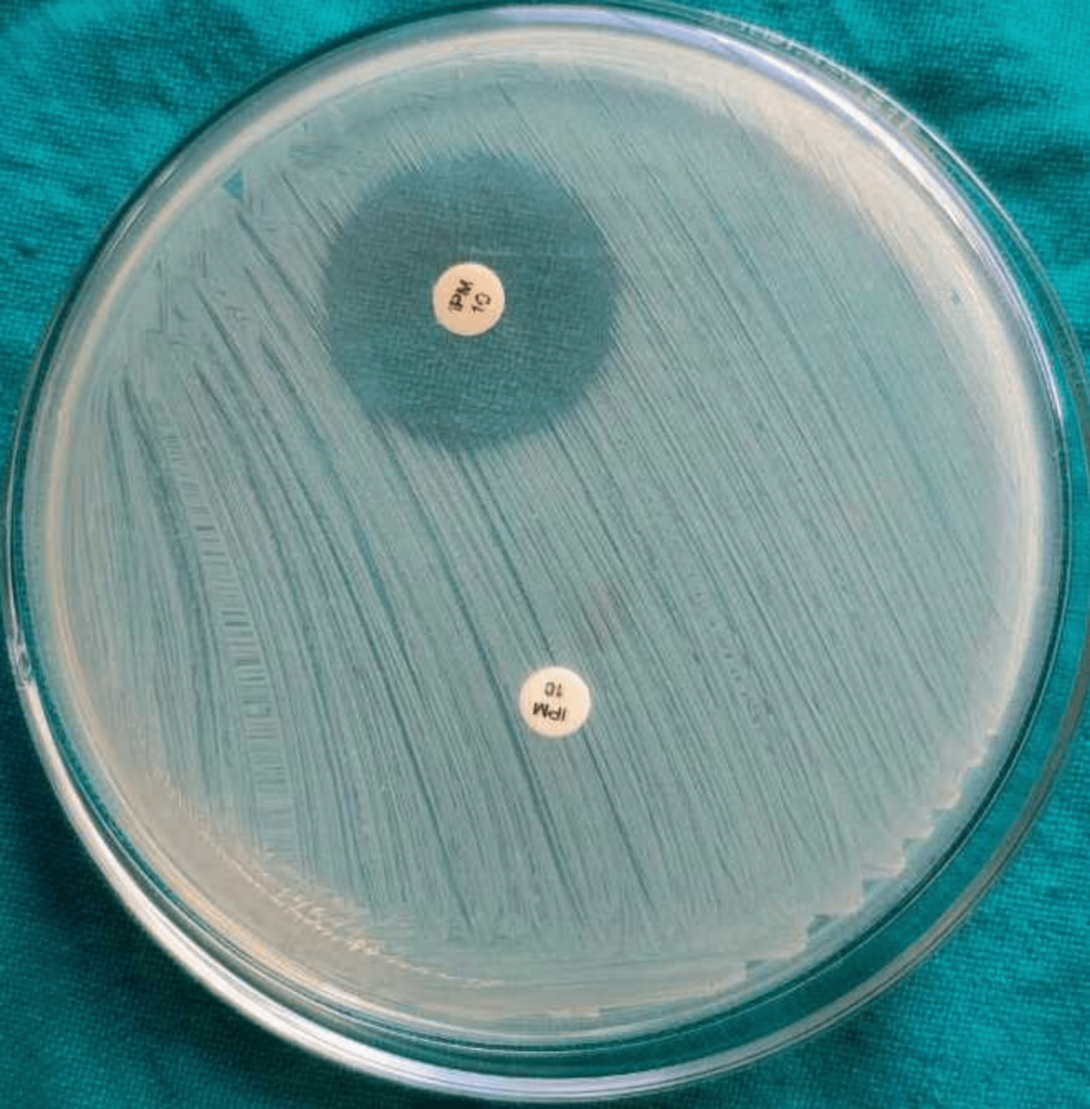
Modified carbapenem inactivation method Positive isolates produce carbapenemase that breaks down carbapenems, leading to reduced antibiotic activity and showing reduced or no zone of inhibition. Negative isolates lack carbapenemase and show enhanced zone size.

**Figure 5:**
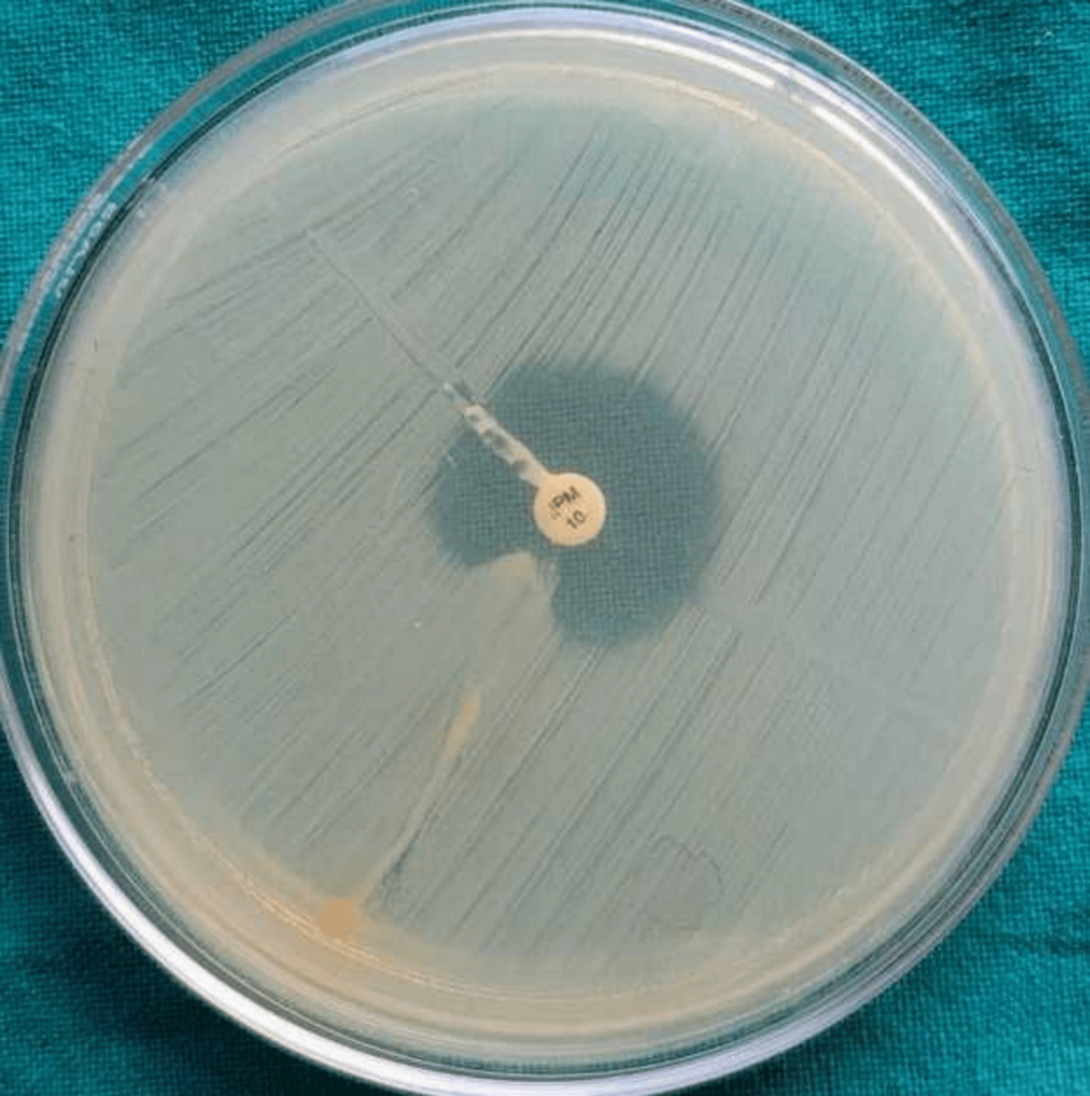
Modified Hodge test Cloverleaf-shaped indentation near the streaked test organism indicates carbapenemase producer.

**Figure 6:**
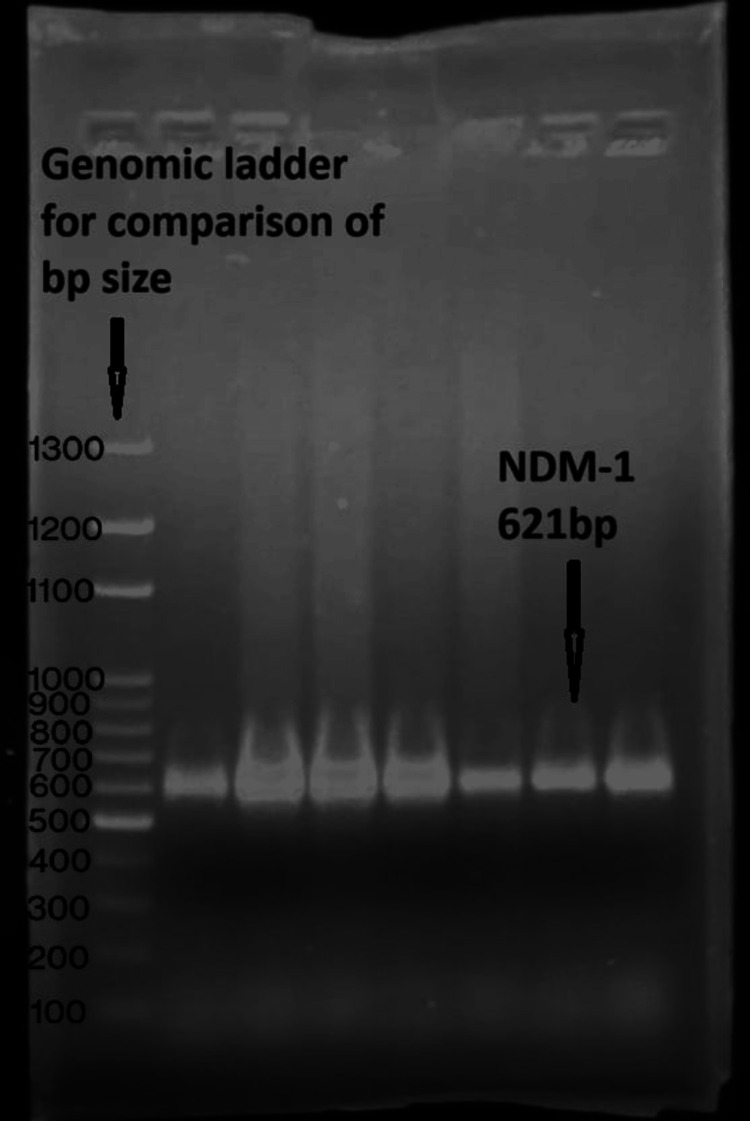
Detection of blaNDM-1 Polymerase chain reaction for the detection of the *bla*NDm-1 gene among CRE isolates, size of 621 bp compared against the genomic ladder. CRE: Carbapenem-resistant *Enterobacterales*

Carbapenemase production in the 47 CRE isolates was assessed using various phenotypic methods. The Rapidec Carba NP and modified Carba NP tests detected carbapenemase activity in 45 isolates (95.74%), identifying 36 *E. coli*, 5* Klebsiella* species, 3 *Citrobacter* species, and 1 *Morganella *species (Figures 1-2). The EDTA combined disk technique identified 39 isolates (83%) as carbapenemase producers (Figure 3), while the modified carbapenem inactivation method (Figure 4) and the modified Hodge test (Figure 5) detected 37 isolates (78.72%) and 33 isolates (70.02%), respectively. Molecular analysis of the *bla*NDM-1 gene, a key marker of carbapenem resistance, revealed that 28 out of 47 CRE isolates (59.6%) carried the gene (Figure 6). Among these, 24 out of 36 *E. coli *isolates (67%) and four out of five* Klebsiella* spp. isolates (80%) were *bla*NDM-1 positive, while *Citrobacter, Morganella*, and *Serratia* species were negative.

The p-values presented in this study (p = 0.007 for phenotypic detection methods and p = 0.003 for *bla*NDM-1 gene detection) indicate a statistically significant association between the presence of carbapenem resistance and the methods used for detection, reinforcing the reliability of these diagnostic tools. These findings underscore the clinical and epidemiological importance of precise and timely identification of CRE, as they provide critical insights for infection control measures, antimicrobial stewardship, and the development of targeted therapeutic strategies.

The total number of urine specimens received was 9235, among which 653 (7%) were considered uropathogens. Out of 653, 555 (85%) were *Enterobacterales, *and 98 (15%) were other than *Enterobacterales*. Of these 555 isolates, 456 were *E. coli*, 56 were* Klebsiella* species, 30 were *Citrobacter* species, 5* were Proteus* species, 5 were *Enterobacter* species, and 1 was *Salmonella, Morganella,* and *Serratia* species (Table 1).

**Table 1:** Distribution of Enterobacterales isolates

Enterobacterales	No. of isolates	Percentage
Escherichia coli	456	82.16
*Klebsiella* species	56	10.09
*Citrobacter* species	30	5.41
*Proteus* species	5	0.90
*Enterobacter* species	5	0.90
*Salmonella* *typhi*	1	0.18
*Serratia *species	1	0.18
*Morganella* species	1	0.18
Total	555	100

Out of a total of 555 *Enterobacterales*, 47 isolates (8.46%) were found to be carbapenem resistant by the Kirby-Bauer disc diffusion method. From the total of 47 carbapenem-resistant isolates, 36 were *E. coli*, 5 were *Klebsiella* species, 4 were *Citrobacter* species, 1 was *Serratia* species, and 1 was *Morganella* species (Table 2).

**Table 2:** Distribution of CRE among Enterobacterales CRE: Carbapenem-resistant *Enterobacterales*

*Enterobacterales *(555)	CRE isolates = 47 (8.46%)
Escherichia coli	36 (7.89%)
*Klebsiella *species	5 (8.93%)
*Citrobacter *species	4 (13.33%)
*Serratia *species	1 (100.0%)
*Morganella *species	1 (100.0%)

Out of 47 carbapenem-resistant isolates, 17 were obtained from the age group of 41 to 60 years, followed by 12 and 11 CRE from the age groups between 61 to 80 years and 21 to 40 years, respectively, whereas a minimum of 3 and 4 CRE isolates were obtained in the two extremes of the age group, i.e., above 81 and 1-20 years, respectively (Table 3).

**Table 3:** Age group distribution of patients having CRE isolates CRE: Carbapenem-resistant *Enterobacterales*

Age group (years)	CRE isolates (47)
0-20	4
21-40	11
41-60	17
61-80	12
More than 81	3

Carbapenemase-producing CRE (CP-CRE) was detected among 47 CRE isolates using the Rapidec Carba test (BioMérieux SA, France), the modified Carba NP test, the Modified Hodge test, the EDTA combined disk technique, and mCIM. The Rapidec Carba NP test and modified Carba NP test detected 45 CP-CRE isolates (95.74%). Among them, 36 were* E. coli*, 5 were *Klebsiella* species, 3 were *Citrobacter* species, and 1 was *Morganella* species, while the EDTA combined disk technique identified 39 isolates (83%) as CP-CRE; among them, 32 were *E. coli*, 4 were *Klebsiella* species, and 3 were *Citrobacter* species. The modified carbapenem inactivation method identified 37 isolates (78.72%) as CP-CRE; among them, *E. *coli were 31, *Klebsiella* species were 4, and *Citrobacter* species were 2. The modified Hodge test identified 33 isolates (70.02%) as CP-CRE; among them, *E. *coli were 28, *Klebsiella* species were 3, and *Citrobacter* species were 2 (Table 4).

**Table 4:** Phenotypic detection of CP-CRE isolates by various methods To determine whether test outcomes were significantly associated with bacterial species, a chi-square test was performed for each method. The Rapidec Carba NP and modified Carba NP tests showed a strong association with species (χ² = 28.59, p < 0.00001), indicating a highly significant difference in detection rates among species. The EDTA combined disc test (χ² = 10.85, p = 0.028) and the modified carbapenem inactivation method (χ² = 10.55, p = 0.032) also showed statistically significant associations, suggesting variability in detection across species. However, the modified Hodge test did not show a significant association (χ² = 6.73, p = 0.151), indicating a more uniform performance across bacterial species. EDTA: ethylenediaminetetraacetic acid; CRE: carbapenem-resistant *Enterobacterales*; CP-CRE: carbapenemase-producing CRE

*Enterobacterales *(555 (CRE = 47))	Rapidec Carba NP (CP-CRE = 45) (95.74%)		Modified Carba NP (CP-CRE = 45) (95.74%)		EDTA combined disc test (CP-CRE = 39) (83%)		Modified carbapenem inactivation method (CP-CRE = 37) (78.72%)		Modified Hodge test (CP-CRE = 33) (70.02%)	
	Po	Ne	Po	Ne	Po	Ne	Po	Ne	Po	Ne
*Escherichia* *coli *(36)	36	0	36	0	32	4	31	5	28	8
*Klebsiella *species (5)	5	0	5	0	4	1	4	1	3	2
*Citrobacter *species (4)	3	1	3	1	3	1	2	2	2	2
*Morganella* species (1)	1	0	1	0	0	1	0	1	0	1
*Serratia* species (1)	0	1	0	1	0	1	0	1	0	1
p-value	The finding was statistically significant as p < 0.05									

A total of 47 CRE isolates were analyzed for the presence of the *bla*NDM-1, a key determinant of carbapenem resistance. Among 36* E. *coli isolates, 24 (67%) were positive for the *bla*NDM-1, while 12 (33%) were negative. Out of five *Klebsiella *species, 4 (80%) were detected with* bla*NDM-1. On the contrary, *Citrobacter* species (four isolates), *Morganella* species, and *Serratia* species (one isolate) were negative for the *bla*NDM-1 gene. Of the total 47 CRE isolates, 28 were positive for* the bla*NDM-1 gene, highlighting the significant presence of CRE, particularly in *E. coli and* *Klebsiella* species, which are known to be major contributors to MDR infections (Table 5).

**Table 5:** Detection of NDM-1 gene among CP-CRE isolates A chi-square test was conducted to assess the association between bacterial species and the presence of the *bla*NDM-1 gene. The test yielded a chi-square statistic of 10.46 with 4 degrees of freedom (df) and a p-value of 0.033. Since the p-value is less than 0.05, the results indicate a statistically significant association between the bacterial species and the presence of the *bla*NDM-1 gene, suggesting that certain bacterial species may be more prone to carrying this resistance gene. CP-CRE: carbapenemase-producing carbapenem-resistant *Enterobacterales*

Isolates (N = 47)	blaNDM-1 gene		
	Total isolates	Positive	Negative
Escherichia coli	36	24 (67%)	12 (33%)
*Klebsiella *species	5	4 (80%)	1 (20%)
*Citrobacter *species	4	0 (0%)	4 (100%)
*Morganella *species	1	0 (0%)	1 (100%)
*Serratia *species	1	0 (0%)	1 (100%)
Total	47	28 (60%)	19 (40%)
p-value	The finding was statistically significant as p < 0.05		

## Discussion

CRE are emerging as significant uropathogens, particularly in UTIs and hospital-acquired infections. Patients with indiscriminate use of antibiotics, on indwelling urinary catheters, recurrent infections, and underlying conditions such as diabetes and immunosuppression are more prone to carbapenem-resistant UTIs. These infections, if left untreated, can further lead to higher morbidity and mortality rates [13]. Uropathogens like *E. coli*, *Klebsiella *spp., and* Enterobacter *spp. pose a major challenge due to their resistance to carbapenems, as they are last-line treatment options. The primary mechanisms of resistance in these bacteria include the production of carbapenemases (VIM, NDM, KPC, IMP, OXA-48 enzymes), which break down carbapenems, rendering them ineffective [14].

Additionally, drug resistance mechanisms like mutation and the overexpression of efflux pumps contribute to reduced antibiotic penetration and increased drug expulsion. The resistance genes are often located on a plasmid, facilitating their rapid spread among bacterial populations. Therefore, our objective was to identify as well as characterize carbapenemase-producing *Enterobacterales *(CPE) isolates using both molecular and phenotypic techniques among uropathogens as recommended by CLSI guidelines [14,15].

In this study, a total of 555 (6.0%) *Enterobacterales *were isolated from 9235 urine specimens. Among 555 *Enterobacterales*, 8.46% were confirmed as CRE isolates. This study was in concordance with Garg et al. (2019), in which the prevalence of CRE isolates was 9.20% among all the *Enterobacterales*. Another study done by Kumar and Mehra (2015) revealed a 6.7% prevalence of CRE isolates among *Enterobacterales *[16,17].

In our study, among 47 CRE isolates, the Rapidec Carba NP test and modified Carba NP test identified 96% CP-CRE-positive isolates. Other phenotypic methods, such as the EDTA combined disc test, MCIM, and MHT, detected 83%, 78.72%, and 70.02% CP-CRE-positive isolates. In concordance with our study, Kour et al. showed 100% CP-CRE-positive isolates by the Rapidec Carba NP and modified Carba NP tests. Another study done by Kumar et al. detected 91.7% CP-CRE isolates by the modified Carba NP method [18,19].

In this study, the EDTA combined disc diffusion test showed 83% CP-CRE isolates. In concordance, a study done by Manju et al. has revealed 82.85% CP-CRE-positive isolates by the same method, which is almost similar to our results. Another study by Bose et al. has shown 100% CP-CRE isolates by the EDTA combined disc diffusion method, which is comparatively higher than our study [20,21].

A study done by Zhou et al. has revealed 99.6% and 88.8% CP-CRE-positive isolates by MCIM and MHT, which is comparatively higher than our study, i.e., 78.72% and 70.02% by both methods [22].

Phenotypically identified CP-CRE isolates underwent further testing for the *bla*NDM-1 gene using PCR. Incorporating genotypic detection, specifically targeting the *bla*NDM-1 gene, enhances diagnostic precision by improving both specificity and sensitivity.

In our study, among 45 CP-CRE isolates, 28 (62%) were detected with the *bla*NDM-1 gene, with the majority found in *E. *coli (23), followed by *Klebsiella* species (4) and *Citrobacter *species (1).

The remaining 17 (38%) isolates, which phenotypically exhibited carbapenemase activity (CP-CRE), were tested negative for the NDM-1 gene, which can be due to the presence of other carbapenemase enzymes such as KPC, IMP, OXA-type, or VIM in the CP-CRE isolates.

A study by Tran et al. conducted in Vietnam also revealed similar findings to our study, where the *bla*NDM-1 gene was the most prevalent (68%) in CP-CRE isolates. Another study by Thapa et al. [23] reported the *bla*NDM-1 gene as predominant in *E. *coli isolates (66.7%) and lower in *Klebsiella* species. Therefore, this study indicates a concern pertaining to the maximum prevalence of the "*bla*NDM-1" gene carrying CRE isolates in uropathogens, aligning with global reports of the increasing dissemination of this resistance gene [23,24].

Limitations of this study

This study has several limitations. First, the sample size is relatively small, which may limit the generalizability of the findings to a larger population. Second, the study was conducted in a single rural teaching hospital, and the results may not reflect the prevalence of *bla*NDM-1 in other regions of Himachal Pradesh or India. Third, molecular characterization was limited to the detection of the *bla*NDM-1 gene, and other resistance mechanisms were not explored. Lastly, clinical outcomes of patients with *bla*NDM-1-positive infections were not assessed, which could provide further insight into the impact of such infections.

## Conclusions

This study highlights the alarming prevalence of CRE among UTI isolates, as our findings reveal 8.46% CRE isolates, with *E. coli* as the most prevalent species. This study further demonstrates the effectiveness of phenotypic methods, such as the Rapidec Carba NP test and modified Carba NP, and genotypic methods in detecting CP-CRE, ultimately confirming the presence of resistance in UTIs. The detection of resistance genes such as *bla*NDM-1 is crucial for guiding targeted therapeutic strategies and preventing outbreaks. Given these findings, we recommend routine implementation of rapid phenotypic and molecular diagnostic techniques in clinical microbiology laboratories to ensure timely and appropriate treatment decisions. Additionally, antimicrobial stewardship programs should prioritize surveillance and judicious use of last-resort antibiotics to mitigate the spread of MDR infections. Future research should explore alternative treatment strategies, including novel antimicrobial agents and combination therapies, to improve patient outcomes in CRE-associated infections.
